# New insight into arginine and tryptophan metabolism in macrophage activation during tuberculosis

**DOI:** 10.3389/fimmu.2024.1363938

**Published:** 2024-04-02

**Authors:** Kangling Zhang, Abhishek Mishra, Chinnaswamy Jagannath

**Affiliations:** ^1^ Department of Pharmacology and Toxicology, University of Texas Medical Branch, Galveston, TX, United States; ^2^ Department of Pathology and Genomic Medicine, Houston Methodist Research Institute, Weill-Cornell Medicine, Houston, TX, United States

**Keywords:** arginine metabolism, tryptophan metabolism, Sirt2, Sirt5, tuberculosis, macrophages

## Abstract

Arginine and tryptophan are pivotal in orchestrating cytokine-driven macrophage polarization and immune activation. Specifically, interferon-gamma (IFN-γ) stimulates inducible nitric oxide synthase (iNOS) expression), leading to the conversion of arginine into citrulline and nitric oxide (NO), while Interleukin-4 (IL4) promotes arginase activation, shifting arginine metabolism toward ornithine. Concomitantly, IFN-γ triggers indoleamine 2,3-dioxygenase 1 (IDO1) and Interleukin-4 induced 1 (IL4i1), resulting in the conversion of tryptophan into kynurenine and indole-3-pyruvic acid. These metabolic pathways are tightly regulated by NAD^+^-dependent sirtuin proteins, with Sirt2 and Sirt5 playing integral roles. In this review, we present novel insights that augment our understanding of the metabolic pathways of arginine and tryptophan following *Mycobacterium tuberculosis* infection, particularly their relevance in macrophage responses. Additionally, we discuss arginine methylation and demethylation and the role of Sirt2 and Sirt5 in regulating tryptophan metabolism and arginine metabolism, potentially driving macrophage polarization.

## Introduction

Understanding how our immune system, particularly macrophages, combats the infection of *Mycobacterium tuberculous* (MTB), the causative agent of tuberculosis, is paramount for developing effective treatments and prevention strategies. Recent findings have highlighted the functional heterogeneity of human M1 and M2 macrophages, with secreted pro-inflammatory and anti-inflammatory cytokines, respectively and exhibit distinct transcriptomes (1). M1 macrophages, driven by interferon-gamma (IFN-γ), are generally associated with the classical activation and pro-inflammatory response. They are characterized by the production of nitric oxide (NO) through inducible nitric oxide synthase (iNOS), which converts arginine to citrulline and NO (2–6). NO has antimicrobial properties and can help in the control of intracellular pathogens like MTB (1, 7–10). Furthermore, M1 macrophages exhibit enhanced autophagy to eliminate MTB (1). In contrast, M2 macrophages, driven by IL-4, are associated with alternative activation and anti-inflammatory response. They express Arginase-1 (Arg1), which converts arginine to ornithine and urea, promoting tissue repair and fibrosis. Furthermore, the production of ornithine in M2 macrophages contributes to immunosuppression, allowing the bacteria or MTB to evade the host immune response. Therefore, in addition to autophagy, the balance between citrulline and ornithine production, determined by arginine metabolism via either iNOS or Arg1, plays a crucial role in the control of MTB infection by polarized M1 vs M2 macrophages. Tryptophan catabolism, up-regulated by indoleamine 2, 3-dioxygenase 1 (IDO1), has been reported in individuals with active TB and latent TB infection (LTBI) (11, 12). Systematic analysis of the gene and protein expression in human alveolar macrophages from MTB-infected individuals identified IL-1β, STAT1 and IDO1 as hub genes associated with MTB growth. Their expression shifts from low initial levels in M1 macrophages to high levels in M2 macrophages. (13). Our proteomics and gene expression analysis further confirmed alterations in tryptophan catabolism between M1 and M2 macrophages and revealed that Interleukin 4 induced 1 (IL4i1) and aryl hydrocarbon receptor (AHR) were down-regulated in M1 macrophages. While arginine and tryptophan are the primary focus of this review, it is important to note that glutamine, serine, and other amino acids are also implicated in immune responses to MTB infection, albeit beyond the scope of this review (14).

**Figure 1:**
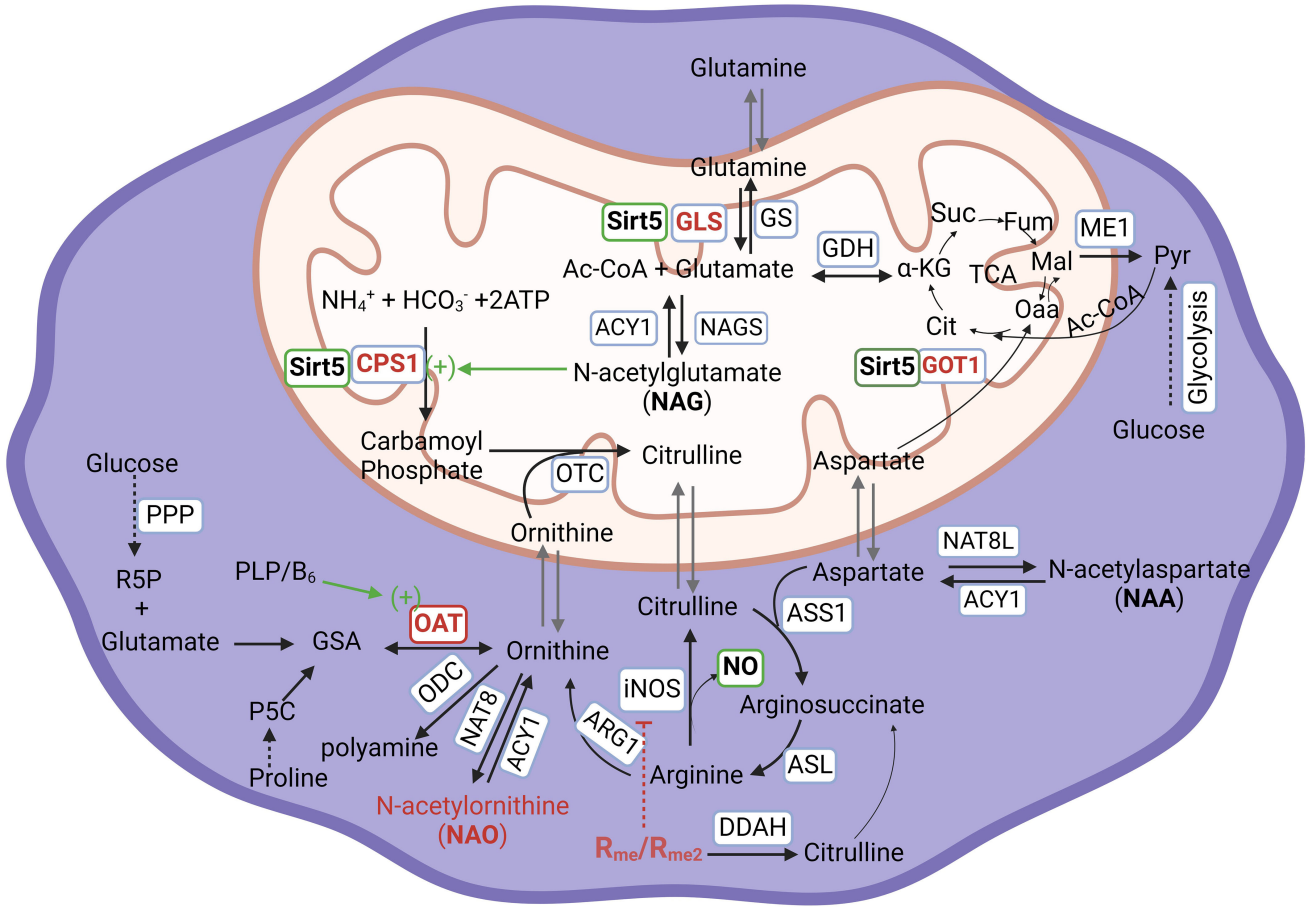
Arginine metabolism and its associated metabolic network in human cells. ACY1, Aminoacylase-1; ARG1, Arginase 1; ASL, Argininosuccinate Lyase; ASNS, Asparagine Synthetase; ASS1, Argininosuccinate Synthase 1, CPS1, Carbamoyl-Phosphate Synthase 1; DDAH, Dimethylarginine Dimethylaminohydrolase; GDH, Glutamate Dehydrogenase; GLS Glutaminase; GOT1, Glutamic-oxaloacetic Transaminase 1; GS, Glutamine Synthetase; ME1, Malic Enzyme 1; NAGS, N-Acetylglutamate Synthase; NAT8, N-Acetyltransferase 8; NAT8L, N-Acetyltransferase 8 Like; iNOS/NOS2:, Inducible Nitric Oxide Synthase/Nitric Oxide Synthase 2; OAT, Ornithine Aminotransferase; ODC, Ornithine Decarboxylase; OTC, Ornithine Transcarbamylas; PRMT, Protein Arginine Methyltransferase; Sirt5, Sirtuin 5. Abbreviation of metabolites, Ac-CoA, Acetyl Coenzyme A; ATP, Adenosine triphosphate; Cit, Citrate; GSA, Glutamyl-γ-semialdehyde; α-KG, α-ketoglutarate; Fum, Fumarate; Mal, Malate; Oaa, Oxaloacetic acid; P5C, Pyrroline-5-carboxylic acid; PLP/B_6_, Pyridoxal 5’-phosphate/Vitamin B6; Pyr, Pyruvate; R5P, Ribose-5-phosphate; R_me_/R_me2_, Arginine monomethylation/Arginine dimethylation; Suc, Succinate; PPP, Pentose Phosphate Pathway; TCA, Tricarboxylic acid cycle. For the purpose of emphasis, enzymes and pathways are in blue-outlined box; Sirt5, active action, and NO are in green-outlined box; Sirt5 substrates, NAO, and methylated arginine are in red-outlined box.

The intricate connection between arginine and tryptophan metabolism in the context of immune cell regulation have been underexplored, despite their shared cytokine regulation. Emerging evidence points to Sirtuin proteins, Sirt5 and Sirt2, as key regulators. Sirt5 influences glutamate synthesis from glutamine through desuccinating glutaminase (GLS) which protects it from ubiquitin-mediated degradation (15). In addition, Sirt5 deacetylates Carbamoyl-Phosphate Synthase 1 (CPS1) and upregulates its activity (16, 17), which enhances the urea cycle, promoting citrulline production from carbamoyl phosphate and ornithine (**Figure 1**). In contrast, Sirt2 appears to regulate tryptophan catabolism through the activation of IL4i. For example, Sirt2 has been implicated in the control of IL4i1 expression in blood cells from acute myeloid leukemia (AML) (18). Recently, we have found that Sirt2 also regulates IL4i1 expression in macrophages, impacting their polarization and control of MTB growth. We observed distinct expression patterns with Sirt5 being more prominent in M1 macrophages compared to M2, and Sirt2 showing higher expression in M2 macrophages compared to M1 (1). These findings suggest that Sirt2 and Sirt5 may differentially regulate arginine and tryptophan metabolism, impacting macrophages polarization and their anti-TB activity (1, 19). This review compiles and synthesizes the latest knowledge on arginine and tryptophan metabolism and their intricate association with MTB infection. The insight offered here are intended to guide future research endeavors, focusing on the expression of Sirtuin proteins in macrophages during tuberculosis. The ultimate aim is to identify potential targets for host-defense-therapy (HDT) against TB.

## Free arginine in immune cells

Arginine occupies a central role in the M1/M2 macrophage dichotomy, which critically determines the fate of invading pathogens, survival or death (20). In M1 macrophages, arginine undergoes conversion into citrulline by NOS/iNOS leading to the release of NO. NO can be further metabolized to reactive nitrogen species (RNS), exhibiting potent bactericidal properties. Conversely, in M2 macrophages, arginine is metabolized into ornithine and urea by arginase (ARG1) (21). Ornithine, in turn, can be utilized for the synthesis of polyamines (putrescine, spermidine, and spermine) through the enzyme ornithine decarboxylase (ODC). Additionally, ornithine can be converted to glutamic γ-semialdehyde and subsequently to proline via ornithine aminotransferase (OAT) (22). These products of ornithine metabolism not only enhance macrophage viability and numbers but also promote the replication of pathogens, including MTB within macrophages. During pathogen infection, monocytes-derived macrophages are initially activated as M1 macrophages, characterized by the release of NO and engagement of autophagy for pathogen clearance. However, as the infection progresses, there is a requirement for macrophages to undergo polarization into M2 macrophages to facilitate continued efferocytosis and resolve inflammation (23). IFN-γ plays a pivotal role as the primary inducer of iNOS/NOS2, orchestrating the transformation of quiescent macrophages into the M1 phenotype. On the other hand, IL-4 or IL-13 serves as a potent stimulus for the expression of ARG1, leading to the transition (polarization) of quiescent macrophages towards the M2 phenotype. This duality in macrophage polarization is crucial in regulating immune response to pathogens. Moreover, the significance of arginine and its metabolism extends to T cell fate and function. When CD4^+^ T cells are activated with CD3 and CD28 antibodies for a duration of 72 hours, there is a noticeable reduction in the levels of arginine, ornithine, and N-acetylornithine compared to their non-activated naïve counterparts (24). This decreased abundance of arginine and ornithine indicates an upsurge in arginine metabolism through ornithine degradation into polyamine and proline. Experimental evidence underscores the essential role of this metabolic shift in T cell activation, differentiation, proliferation, survival, and to anti-tumor activity (24).

Host cells, particularly macrophages, have a critical need for arginine to support their growth and function. Arginine can be obtained from extracellular nutrition or synthesized *de novo* from citrulline. The process of *de novo* biosynthesis extends to involve glutamine metabolism. Glutamine undergoes deamination to form glutamate through the action of glutamate synthase (GLS). Subsequently, glutamate is acetylated to produce N-acetylglutamate (NAG) in the presence of acetyl-CoA and glutamine acetyltransferase (NAGS). Notably, the Sirtuin protein Sirt5 is intricately involved in this metabolic pathway. Sirt5 has been reported to regulate ammonia detoxification through deacetylation, consequently activating carbamoyl phosphate synthetase 1 (CPS1) in the liver (17, 25–27). NAG plays an important role in the activation of activates CPS1, which catalyzes the conversion of ammonia into carbamoyl phosphate. This enzymatic step represents the initial stage of the urea cycle (**Figure 1**). Carbamoyl phosphate further participates in two significant pathways: 1. It reacts with ornithine to synthesize citrulline. Subsequently, citrulline serves as a precursor for arginosuccinate and, ultimately, the production of arginine; 2. It can also directly react with aspartate to form carbamoyl aspartate which is further utilized for pyrimidine synthesis, contributing to the formation of essential nucleotide components (28). Sirt5’s role in regulating ammonia production extends beyond the liver and includes non-liver cells. In non-liver cells, Sirt5 desuccinylates mitochondria GLS. However, it is important to note that two studies have reported contradictory results regarding the impact of Sirt5 on GLS activity: In one study, Sirt5 was shown to desuccinylate Lys245 and Lys320 of GLS. This desuccinylation by Sirt5 inhibits the activity of GLS, resulting in a reduced conversion of glutamine into glutamate, leading to a decrease in ammonia production (29). In contrast, in another study, Sirt5 was found to mediate desuccinylation of Lys164 of GLS, leading to the protection of GLS from ubiquitation. This stabilization of GLS by Sirt5 promoted the conversion of glutamine into glutamate and, consequently, an increase in ammonia production (15). The conflicting outcomes from these two studies indeed highlight the complexity of Sirt5’s role in regulating glutamine catabolism, ammonia production, and detoxification. These discrepancies suggest that our current understanding of Sirt5’s mechanisms in these processes is incomplete, and additional, as yet unidentified, mechanisms could be at play. It is likely that the multifaceted roles of Sirt5 involve a network of interaction and dependencies that necessitate more comprehensive investigation. Indeed, a recent research sheds light on Sirt5’s involvement in cancer cell development. In pancreatic ductal adenocarcinoma cells (PDAC), the downregulation of Sirt5 or its knock-down led to an increase in the activity of glutamate oxaloacetate transaminase 1 (GOT1) through acetylation (30). GOT1, a cytosolic aspartate aminotransferase, is responsible for converting aspartate derived from glutamine into oxaloacetate and subsequently into malate for the tricarboxylic acid (TCA) cycle or for the synthesis of pyruvate which is the end product of glycolysis (**Figure 1**). Notably, reduced Sirt5 expression in PDAC cells promoted cancer cell proliferation, whereas enhancing Sirt5 expression or activating it through a SIRT5 activator, MC3138, restrained tumor growth (30). In an alternate report, PDAC expressed a preference for the *de novo* synthesis (DNS) of ornithine from glutamate, facilitated by ornithine aminotransferase (OAT), as opposed to the traditional biosynthesis from arginine (31). However, it remains unknown whether the depletion or inhibition of Sirt5 leads to increased acetylation or succinylation, subsequently stabilizing OAT. Additionally, it’s unclear whether the Sirt5-GOT1 axis activates an alternative metabolic pathway or influences the pentose-phosphate pathway (PPP) after decoupling glycolysis with aspartate metabolism towards pyruvate, which subsequently influences the synthesis of pyridoxal phosphate (PLP). It is worth noting that PLP, an active form of vitamin B6, is vital as a cofactor for OAT and can be synthesized from ribose-5-phosphate at least in bacteria (**Figure 1**) (32, 33). In human macrophages and naïve CD4^+^ T cells, there is a significant presence of N-acetyl-ornithine (NAO), and its concentration is markedly decreased in activated T cells (12) but increased in non-MTB infected M1 macrophages (19, 24) and Sirt5 depleted THP-1 cells (our unpublished data). The origin of increased NAO and its potential association with elevated ornithine through DNS is not well-established. The exact correlation between Sirt5 expression and NAO formation in immune cells is a subject that necessitates further investigation. This includes understanding their role in arginine synthesis and metabolism, and how these processes are interconnected with glutamine metabolism mediated by Sirt5.

## Arginine is essential for survival and killing of MTB

Arginine serves a dual role in the context of MTB infection. It is not only essential for host macrophage development but also plays a crucial role in the survival or elimination of MTB through autocrine-paracrine cytokine signaling pathways (34). There are multiple mechanisms through which MTB can acquire arginine: 1. *Direct Uptake*. MTB is predicted to have up to five putative arginine transporters, suggesting that the bacterium can directly absorb arginine from the host (35). *In vitro* models show that MTB utilizes arginine both as a nitrogen and carbon source with the involvement of Rv2323c which comprises 909 base-pairs and has been annotated as a dimethylarginine dimethylaminohydrolase (27). Rv2323c is responsible for synthesizing ornithine, proline, and other amino acids through mechanisms that are yet to be fully defined (36). Additionally, Rv2323c also functions as an arginine demethylase, potentially being secreted into host cells to hydrolyze methylated arginine (36). The dependence of MTB on arginine is underscored by its inability to survive in arginine-depleted medium (37). This is further supported by the observation that an arginine auxotrophy MTB strain was unable to persist in a mouse infection model (38), indicating that obtaining arginine directly from the medium might be insufficient for meeting MTB’s high growth demand when its own arginine synthesis is blocked (39). The fine-tuned balance of arginine availability and distribution within host cells and MTB appears to be a crucial factor in determining pathogen survivability. In the host cells, like macrophages, arginine is supplied as a part of amino acid nutrition to pathogens such as MTB, supporting growth and survival. Concurrently, the host cells utilize arginine to produce NO for pathogen elimination. Even in the absence of NO, arginine can still contribute to pathogen clearance by increasing macrophage numbers and viability (40). Given this complex interplay, arginine has been considered as a potential adjunctive therapy in the context of active tuberculosis (41). Pilot studies are exploring the safety and efficacy of supplemental arginine to enhance immune function in individuals with HIV/AIDS (42). These findings highlight the multifaceted role of arginine in host-pathogen interactions and suggest its therapeutic potential for combating infectious diseases.

**Figure 2:**
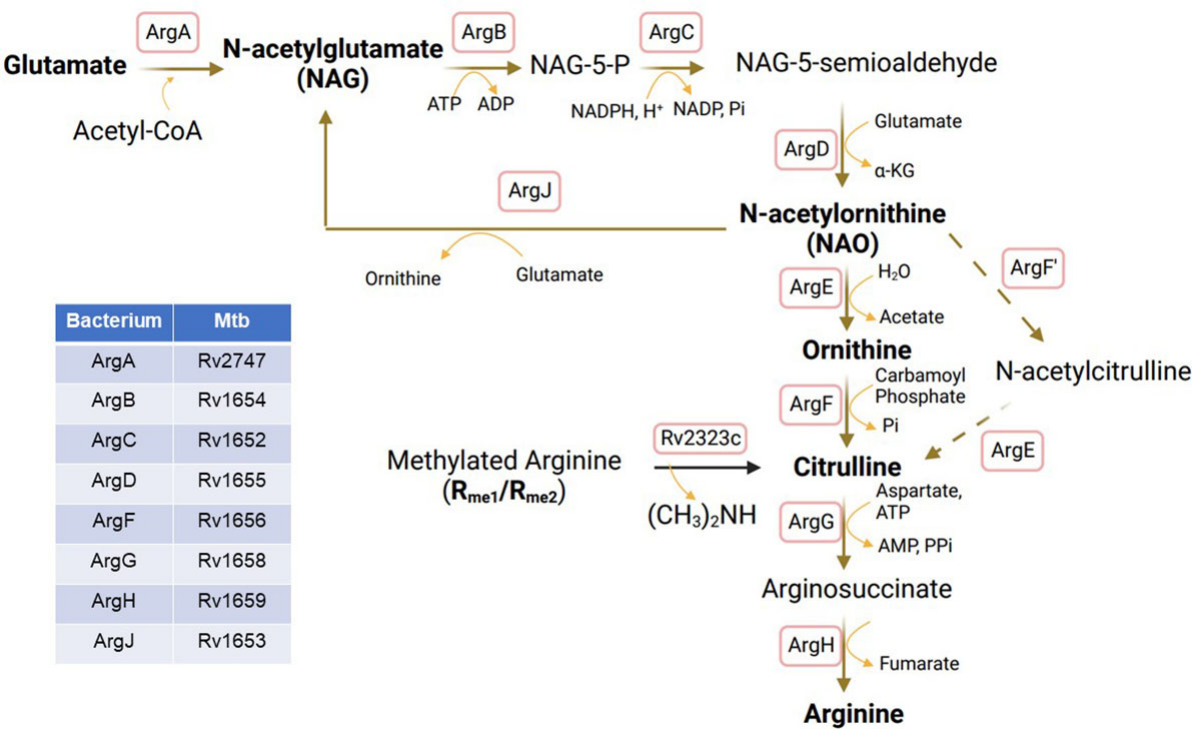
Arginine biosynthesis from glutamate in MTB. ArgA, glutamate acetyltransferase/N-acetylglutamate synthetase; ArgB, Acetylglutamate kinase; ArgC, N-acetyl-γ-glutamyl-phosphate reductase; ArgD, Acetyornithine aminotransferase; ArgE, N-acetylornithine deacetylase; ArgF, Ornithine carbamoyltransferase; ArgF’, Acetylornithine transcarbamylase; ArgG, Arginosuccinate synthase; ArgH, Arginosuccinate lyase; ArgJ, Glutamate N-acetyltransferase.

2. *Synthesis from glutamine/glutamate*. Majority of arginine required by MTB is synthesized *de novo* from glutamate through a unique route known as the glutamate - glutamyl-γ-aldehyde – ornithine – citrulline pathway (**Figure 2**). This pathway is specific to bacteria and plants and is not believed to exist in human or animal cells (43). The pathway involves the action of eight different enzymes, including ArgA-H and ArgJ (**Figure 2**). The synthesis process begins with the acetylation of glutamate to form NAG by glutamate acetyltransferase/N-acetylglutamate synthetase (ArgA) in the presence of acetyl-CoA. NAG is then phosphorylated at the carbonyl group, leading to the formation of NAG-5-phosphate with the help of N-acetylglutamate kinase (ArgB). ArgC subsequently reduces NAG-5-phosphate to form NAG-5-semioaldehyde, which serves as an intermediate in the synthesis of NAO through the action of acetylornithine aminotransferase (ArgD). NAO is then deacetylated by ArgE to form ornithine. Ornithine further reacts with carbamoyl phosphate to yield citrulline, a process catalyzed by ArgF, which is the bacterial equivalent of human ornithine transcarbamylase (OTC). Additionally, citrulline can be synthesized from acetylcitrulline by transferring acetyl groups from NAO, involving the enzymes ArgF’ and ArgE. It is worth noting that an analog of ArgE, an ornithine deacetylase (hydrolase) is known to exist in E. Coli and plants (44, 45), although its presence in MTB has not yet been identified (46, 47). However, it is likely that MTB possesses an enzyme with similar functions, enabling the completion of this unique arginine biosynthesis pathway. Citrulline undergoes two steps of metabolic conversions to become arginine. Initially, it is transformed into arginosuccinate by ArgG, an enzyme with similarities to Argininosuccinate Synthase 1 (ASS1) in human cells (**Figure 1**). Subsequently, arginosuccinate is further metabolized into arginine by argininosuccinate lyase, specifically ArgH (the human equivalent being ASL (**Figure 1**).

The biosynthesis of arginine from NAG in MTB, as well as in other bacteria and plants, is unique to these organisms and presents a potential target for the development of anti-TB drugs. The first trial involved Pranlukast (PRK), which acts as a non-competitive inhibitor with a K_i_ value of 139 μM against MTB-specific ornithine acetyltransferase, ArgJ, by targeting its surface large pocket instead of substrate-binding sites, thereby reducing cross-reaction with other proteins that may also have the same substrate-binding sites. Using a microplate Alamar blue assay, the minimum inhibitory concentration of PRK against MTB H37Rv is 5 µg/mL (39). Another compound, Sorafinib (SRB) was also identified as a non-competitive inhibitor with a K_i_ value of 244 μM against ArgJ, targeting the same pocket (48). The minimum inhibitory concentration of SRB against MTB H37Rv is 10 µg/mL. It seems PRK has higher efficacy than SRB. PRK has demonstrated the ability to inhibit the growth of MTB H37Rv at the micromolar level and has shown effectiveness in killing when used in combination with standard anti-tuberculosis drugs such as rifampicin and isoniazid *in vitro* (48). It shows that PRK inhibits the survival of MTB in macrophage infection model without affecting the host cells and the inhibitory effect is compromised by supplemental arginine. Furthermore, PRK treatment with the dose of 40 mg/kg body weight has been found to induce bacterial killing and reduce granuloma formation in the lungs of MTB-infected mice (48). These findings highlight the potential of targeting this unique arginine biosynthesis pathway for developing anti-TB drugs.

3. *Synthesized from methylated arginine*. Arginine in MTB can also be synthesized through the cleavage of methylated arginine, resulting in the formation of citrulline. Citrulline is subsequently metabolized to arginosuccinate and, finally, converted into arginine (**Figure 1**). Interestingly, Rv2323c, cited above serves as a dimethylarginine dimethylaminohydrolase (DDAH) or arginine demethylase that cleaves methylated arginine into dimethylamine and citrulline. Following the cleavage by Rv2323c, citrulline is processed as previously described, first metabolized into arginosuccinate by ArgG then into arginine by ArgH (**Figure 2**). The availability of arginine in MTB is also subject to regulation by the dormancy survival regulator regulon (DosR) proteins, which consist of 48 co-regulated components. DosR binding to the promoter region of ArgC regulates arginine synthesis, particularly during the anaerobic dormancy phase of persistent MTB (49, 50). This regulatory mechanism adds another layer of complexity to arginine metabolism in MTB and its role in survival strategies.

## Arginine methylation

Arginine methylation is a post-translational modification that occurs in proteins and catalyzed by enzymes known as protein arginine methyltransferases (PRMTs). There are currently nine known PRMTs, labeled as PRMT1 to PRMT9 with PRMT4 having another name as CARM1, each with distinct abilities to methylate arginine in various forms. These forms include mono-methylarginine (MMA or Rme1), symmetric omega-N^G^, N^G^-di-methylarginine (SDMA or Rme2s), and asymmetric omega-N^G^, N^G^-di-methylarginine (ADMA or Rme2a). MMA involves the addition of a single methyl group to one of the terminal nitrogen atoms of the guanidino group. SDMA incorporates two methyl groups symmetrically placed on each terminal nitrogen atom of the guanidino groups. In the case of ADMA, two methyl groups are added to the same terminal nitrogen atom of the guanidino group. Among these forms, Rme2a (ADMA) is the predominant form, with Rme1 (MMA) and Rme2s (SDMA) levels typically ranging from 20% to 50% of that of Rme2a (ADMA) (51). Thus, methylation plays a role in regulating various cellular processes and protein functions in both health and disease.

The nine PRMT members can be primarily categorized into three types based on their structural differences and the final forms of methylation products they generate. Type I PRMTs, including PRMT1, 2, 3, 4, 6 and 8, are responsible for catalyzing the formation of Rme1 and Rme2a (52). Type II PRMTs, comprising PRMT5 and 9, are involved in generating Rme1 and Rme2s (53–55). Type III PRMT, represented by PRMT7, can only produce Rme1 (52, 56). Arginine methylation in the human proteome primarily occurs within glycine-arginine rich and proline-glycine-methionine-rich regions. It is frequently found in histones and a plethora of RNA-binding proteins (51). Heteregeneous nuclear ribonucleoproteins (hnRNPs) constitutes a significant portion of the total arginine methylation within the cell nucleus, accounting for about 65% of such modification (57, 58). Quantitatively comparable to protein phosphorylation, about 0.5%–2% of arginine residues are methylated in mammalian cells and tissues (59–61). These ost-translational modifications play a role in a number of different cellular processes, including the maintenance of genome integrity, regulation of cell cycle and transcription, RNA splicing and metabolism, RNA-protein interactions, DNA damage response and cancer-related processes (62–68). Therefore, arginine methylation contributes significantly to the regulation and coordination of numerous critical cellular functions.

## Methylation of free arginine

Upon the proteolytic cleavage of arginine-methylated proteins, free intracellular mono-methylated arginine (MMA), symmetric di-methylated arginine (SDMA), or asymmetric di-methylated arginine (ADMA) are generated. These methylated arginines can be transported into the extracellular space, including plasma, directly affecting the concentrations of methylarginines in the plasma. The body clears free methylarginines by renal excretion or hepatic metabolism. In addition, MMA and ADMA, but not SDMA, can be degraded by a class of intracellular enzymes known as dimethylarginine dimethylaminohydrolases (DDAH). Plasma and urine levels of arginine and methylated arginines have been measured in healthy children, revealing concentrations of 52.2-127.7 μM for arginine, 0.06-0.16 μM for MMA, 0.42-1.10 μM for ADMA, 0.41-0.96 μM for SDMA (69). However, the concentrations of these compounds are notably higher in peripheral blood mononuclear cells (PBMC) (70, 71). It is worth mentioning that a disproportionately high amount of ADMA has been observed in human monocytes and macrophages (19). To date, a *de novo* synthetic pathway for generating MMA, ADMA, or SDMA from free arginine has not been identified. Therefore, it is believed that free methylarginines found in both the plasma and within cells are derived solely from the degradation of proteins containing methylated arginines (72). Additionally, the turnover and degradation rates of proteins are assumed to be higher in PBMC and its derivatives compared to the plasm, leading to the accumulation of methylated arginines.

## Free methylated arginine in immune cells during tuberculosis

Mono-methylated arginine (MMA) is well-known inhibitor of inducible nitric oxide synthase (iNOS) and has been extensively used for studying NO’s mechanistic roles during macrophage activation, cardiovascular diseases, hypercholesterolemia, nervous system disorders, lung diseases, autoimmunity, and viral/bacterial infections, including tuberculosis (7, 73–76). ADMA is considered as a nonspecific competitive inhibitor of nitric oxide synthase (NOS) and a weak inhibitor of iNOS (77, 78). SDMA, on the other hand, does not have NOS inhibitory activity. Both NMA and ADMA can be recycled back to arginine within the arginine metabolic pathway. Initially, they are hydrolyzed by dimethylarginine dimethylaminohydrolose (DDAH) to produce citrulline along with either mono- or dimethylamine. Subsequently, arginine is synthesized from citrulline through a two-steps enzymatic process involving the enzymes argininosuccinate synthase 1(ASS1) and argininosuccinate lyase (ASL) (**Figure 1**). Enhanced hydrolysis of MMA and ADMA not only alleviates the suppression of iNOS, promoting the production of antibacterial NO, but also generates additional arginine from citrulline, the hydrolytic product of MMA and ADMA, which can strengthen host defenses against mycobacterial infections (79). In activated macrophages, high concentration of ADMA, and possibly MMA, block NO synthesis, resulting in increased expression of the receptor for oxidized LDL (oxLDL), also known as lectin-like oxLDL receptor (LOX-1). This can contribute to lipidosis and foam cell formation (80).

Studies show that oxLDL supports MTB survival in macrophages by impairing lysosomal function. Treatment of macrophages with oxLDL results in the accumulation of lysosomal cholesterol, altered levels of lysosomal and autophagy markers (such as LAMP1, LAMP2, Cathepsin D, and L), reduced MTB colocalization with lysosomes, and an increased bacterial load (81). Further, plasma oxLDL levels are significantly elevated in type 2 diabetes mellitus (DM) and they are associated with high levels of triglyceride in diabetes-associated tuberculosis (DMTB) patients, suggesting oxLDL may serve as a risk factor for DMTB (81). During MTB infection, the uptake and accumulation of oxLDL in macrophages are facilitated by proteins such as CD36 and COX-1 (82). Interestingly, the deletion of LOX1 has been found to improve the neutrophil response, enhance bacterial clearance, and reduce lung injury in mice with polymicrobial sepsis (83). While this study was conducted using a polymicrobial model and mice, it provides insights into potential mechanisms relevant to pathogenesis of tuberculosis. Moreover, there is a positive association between the ADMA-oxLDL-LOX-1 axis and the severity of bacterial infections. ADMA appears to drive macrophages towards an M2-like subtype characterized by increased expression of markers such as Arg1, CD163, and CD206, while suppressing IL-10 and dectin-1 expression, ultimately impairing phagocytosis (84). Therefore, the removal of ADMA and MMA from host cells via DDAH activation may be beneficial in promoting M1 polarization and enhancing immune protection.

The role of the MTB DDAH paralog protein, Rv2323c, presents an intriguing paradox. On one hand, it is involved in producing arginine and other products derived from arginine metabolism, which are essential for bacterial growth and survival (36). However, it also has a second role: Rv2323c is secreted by MTB into the host environment, where it hydrolyzes methylated arginine. This unique function enhances the host’s immune response, potentially contributing to self-destruction of MTB by M1-macropnages as described above. This dual role suggests that Rv2323c may serve as a strategy employed by MTB to modulate the host immune response. By hydrolyzing methylated arginine, Rv2323c could contribute to a more robust immune defense within the host. It may reflect the complex relationship between MTB and the host’s immune system, where the pathogen both relies on specific nutrients for survival and triggering immune responses that could be detrimental to its persistence. The detailed mechanisms of Rv2323c’s and their impact on MTB pathogenesis and host defense are the subject of ongoing research.

## Tryptophan catabolism in immune cells

**Figure 3:**
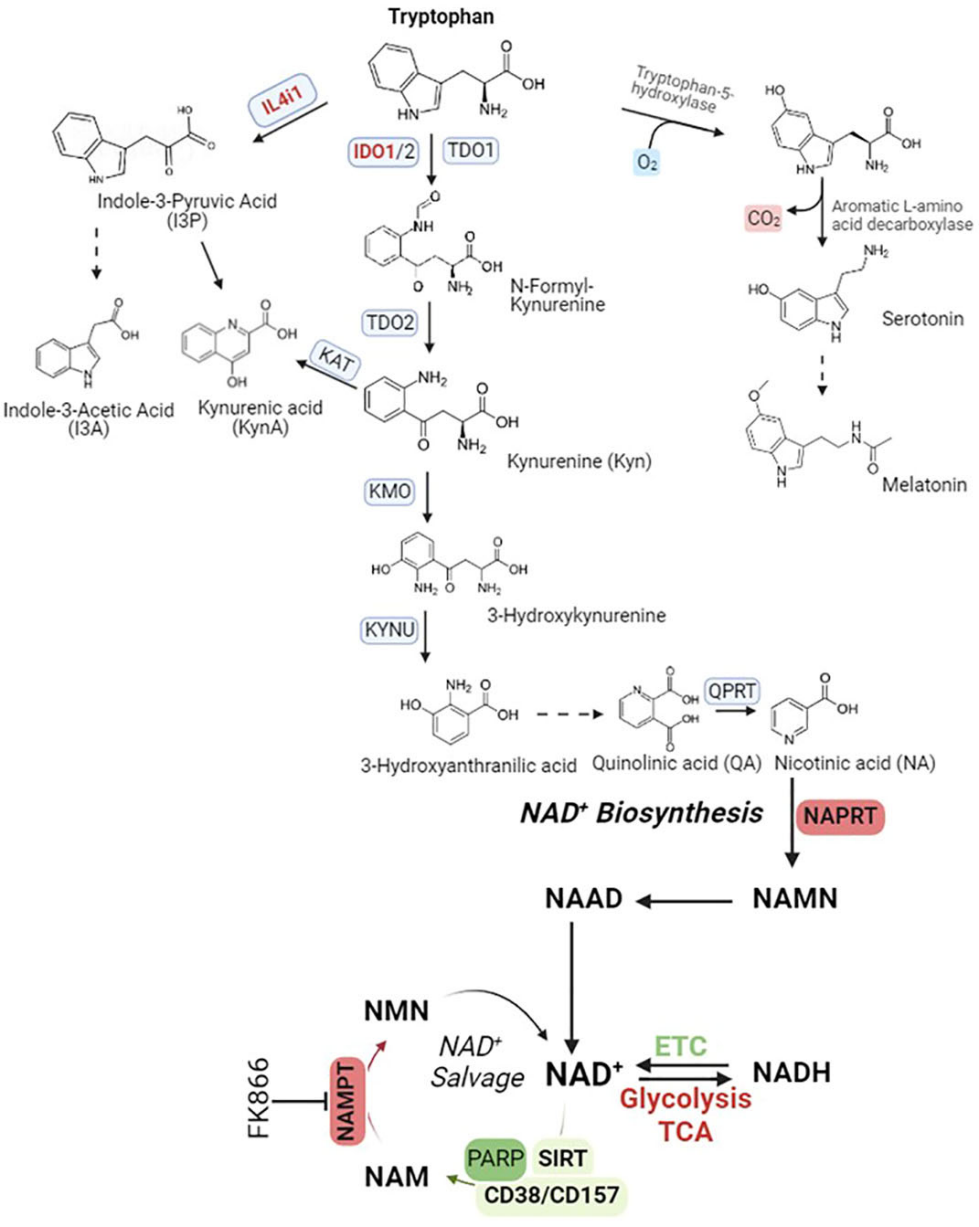
Tryptophan catabolism pathways. IL4i1, Interleukin 4 Induced 1; IDO1, Indoleamine 2, 3-Dioxygenase 1, TDO2, Tryptophan 2,3-Dioxygenase; KAT, Kynurenine Aminotransferase; KMO, Kynurenine 3-Monooxygenase; KYNU, Kynureninase; QPRT, Quinolinate Phosphoribosyltransferase; NAMPT, Nicotinamide Phosphoribosyltransferase; NAPRT, Nicotinate Phosphoribosyltransferase; PARP, Poly(ADP-Ribose) Polymerase 1; SIRT, Sirtuin; CD38, Cluster Of Differentiation 38/ADP-Ribosyl Cyclase 1; CD157, Cluster Of Differentiation 157/Bone marrow stromal cell antigen 1 (Bst1)/ADP-Ribosyl Cyclase 2. Abbreviation of metabolites in NAD^+^ salvage pathway, NAD^+^, Nicotinamide adenine dinucleotide; NADH, NAD + Hydrogen (NAD reduced form); NAAD, Nicotinic acid adenine dinucleotide; NAM, Nicotinamide; NAMN, Nicotinic acid mononucleotide; NMN, Nicotinamide mononucleotide.

Tryptophan catabolism comprises of at least three metabolic pathways, tryptophan IDO1-oxidation pathway, tryptophan IL4I1-oxidation pathway, and tryptophan serotonin pathway (**Figure 3**).

Tryptophan IDO1-oxidation pathway: While tryptophan is an indispensable component for protein synthesis and various biological processes, it is intriguing to note that a significant portion of dietary tryptophan embarks on an alternative journey. This path leads to the formation of an array of downstream metabolites, primarily orchestrated by the kynurenine pathway. This pathway initiates with the pivotal conversion of tryptophan into N-formylkynurenine, a step governed by three distinct enzyme isoforms: indoleamine 2,3-dioxygenase 1 (IDO1), indoleamine 2,3-dioxygenase 2 (IDO2), and tryptophan 2,3-dioxygenase (TDO). Among the three enzymes responsible for catalyzing the conversion of tryptophan into N-formylkynurenine, IDO1 stands out as a captivating subject. Due to its conformational plasticity and adaptability to a complex and highly regulated catalytic activity, IDO1 has a remarkable ability to orchestrate changes in expression profile of immune cells towards a highly immunoregulatory phenotype (85).

The expression of IDO1 is primarily initiated by IFN-γ, but it is interesting to note that IFN-γ, which induces NO production in M1 macrophages, can also inhibit IDO1 expression through a feedback-loop mechanism (86). An essential end product of this pathway is quinolinic acid (QA), which serves as a precursor for nicotinic acid (NA). The conversion of QA to NA is facilitated by quinolinate phosphoribosyl transferase (QPRT). Subsequently, NA undergoes a series of conversions into nicotinamide (NAMN) and finally into NAD^+^. The transformation of NAD^+^ to NADH, a redox reaction, is required for the function of numerous dehydrogenases involved in the energy-associated metabolic pathways of living cells (87). For instance, NAD^+^ serves as an essential cofactor for the functioning of enzymes like glyceraldehyde phosphate dehydrogenase (GAPDH) in glycolysis. Additionally, it is involved in the oxidative decarboxylation of pyruvate into acetyl-CoA through the activity of pyruvate dehydrogenase (PDH). NAD^+^ also participates in the conversion of lactate to pyruvate, a process mediated by lactate dehydrogenase (LDH). Furthermore, it is indispensable for the proper functioning of enzymes such as α-ketoglutarate dehydrogenase, isocitrate dehydrogenase, and malate dehydrogenase in the tricarboxylic acid (TCA) cycle. Beyond that, it plays a crucial role in the β-oxidation of fatty acids, facilitated by 3-hydroxyacyl-CoA dehydrogenase. Lastly, NAD^+^ is involved in alcohol metabolism, serving as a cofactor for aldehyde dehydrogenase enzymes (87, 88). Furthermore, NAD^+^ serves as the exclusive cofactor for the type III histone/protein deacetylase Sirtuin proteins (Sirt1-7). NAD^+^ is also actively involved in other essential processes, including its role in PARPs (poly(ADP-ribose) polymerase) during DNA repair and the function of CD38 and its analog CD157 in cyclic ADP-ribose (cADPR) synthase. To maintain NAD^+^ homeostasis, it can be replenished from the oxidation of NADH via the electron-respiration chain, *de novo* biosynthesis from tryptophan catabolism, biosynthesis from nicotinic acid (NA) through the Preiss-Handler pathway, and the salvage pathway (87).

In macrophage stimulated by lipopolysaccharide (LPS) alone or in combination with IFN-γ, intracellular NAD^+^ levels decrease because of an elevated ROS-induced DNA damage response and subsequent repair through PARP activation (89). To counterbalance this decrease, cells initiate a compensatory response by increasing the protein expression of nicotinamide phosphoribosysyltransferase (NAMPT), the rate-limiting enzyme in the NAD^+^ salvage pathway (89). This rescue strategy for NAD^+^ utilization and resupply are critical in maintaining glycolysis and inflammation. In inflammatory and ageing macrophages, the *de novo* NAD^+^ biosynthesis pathway through tryptophan catabolism is activated due to elevated expression of IDO1, which helps to maintain NAD^+^ homeostasis and restrict inflammation. Inhibition or knockdown of IDO1 or other enzymes in this pathway, such as QPRT, results in the suppression of mitochondrial NAD^+^-dependent signaling and NAD^+^ synthesis from the electron transport/respiration chain (ETC), increased glycolysis, and impaired resolution of inflammation. However, the NAD^+^ reduction resulting from the inhibition of tryptophan catabolism can also be rescued by increased NAD^+^ production through NAMPT or nicotinate phosphoribosyltransferase (NAPRT) (**Figure 3**). These enzymes serve as the biomarkers of chronic and acute inflammatory diseases (90). In the context of resolving inflammation, inhibiting NAMPT using FK866 appears to be a promising strategy for the treatment of inflammatory diseases. However, it is important to note that NAMPT-mediated glycolysis is also required for the antitumor immunity of tumor-infiltrating macrophages. In this scenario, STAT1 binds to the STAT1 binding elements, also known as NAMPT-Regulatory Element-1 (NRE1), located in the first intron of NAMPT. This binding enhances the expression of NAMPT, leading to aerobic glycolysis that promotes the expression of pro-inflammatory genes in tumor-associated macrophages (TAM) stimulated with IFN-γ (91). Inhibition of NAMPT results in NAD^+^ depletion and impairs phagocytosis, as demonstrated by a reduced intracellular uptake of FITC-labeled, complement-opsonized zymosan (COZ particles) in RAW 264.7 macrophages treated with FK866 (92).

Tryptophan IL4i1-oxidation pathway: IL-4i1 is an L-amino acid oxidase (LAAO) that primarily catalyzes the oxidation of phenylalanine and tyrosine (93, 94) and can catalyze the oxidation of tryptophan, albeit with reduced efficacy (95). Initially, identified as an early IL-4-inducible gene in B cells, IL4i1 is expressed in primary macrophages, and dendritic cells, and tumor-associated macrophages (93, 96–99). The expression of IL4i1 seems to be involved in phagocytic processes, as IL4i1^+^ macrophage population coexists with the genes involved in phagosome maturation in the tumor microenvironment (TME) (100). This observation is further supported by the finding that highly phagocytic macrophages expressing IL4i1 play a role in the removal of apoptotic B cells (93, 99). Single-cell RNA sequencing (scRNAseq) has revealed overlapping expression of IL4i1 and IDO in the TME and in myeloid cells, particularly LAMP3^+^ cells in the TME (101, 102). Since the gene expression of IDO1 is stimulated by IFN-γ and TNF-α (103), it is reasonable to speculate that the gene expression of IL4i1 is also activated in cells stimulated by IFN-γ and/or TNF-α. Consistent with this, we found both IDO1 and IL4I1 gene expression were upregulated in M1 macrophages exposed to MTB (1). However, proteomics analysis indicated that the protein expressions of both IDO1 and IL-4i1, along with AHR, were reduced compared to IL-4-treated M2 macrophages. In this context, it was not surprising to observe that overexpression of IL4il could drive the expression of M2 macrophage markers, such as Fizz-1 and Arg1, while inhibiting the cytokines typical of M1 macrophages, including IL-1β and TNF-α (104). Accordingly, additional research is necessary to elucidate the disparity between RNA gene expression and protein expression and their respective roles in regulating macrophage polarization. The products of IL4i1-catabolized tryptophan oxidation, including indole-3-pyruvic acid (I-3-P), indole-3-acetic acid (I-3-A), and kynurenic aid (KynA), are AHR agonists (105). The work of Sadik et al. further suggests that IL4i1 activates the expression of AHR, IDO, and TDO2, which collectively suppress tumor immunity in the hypoxic TME of glioblastoma derived GBM cells, thereby promoting cancer progression (105). The suppressive effect exerted by IL4i1 on immune cells is also evident by an enrichment of myeloid-derived suppressor cells (MDSCs) and Foxp3+ regulatory T (Treg) cells, a characteristic frequently observed in chronic lymphoma leukemia (CLL) (105). In addition, the mTORC1 pathway is inhibited by IL4I1 in activated naïve CD4^+^ T cells (106). Furthermore, IL4i1 plays a crucial role in regulating antigen-presenting cell (APC)-mediated inflammatory responses during acute and chronic MTB infection. This is supported by data that IL4i1–deficient (IL-4i1^−/−^) mice display enhanced protection against acute MTB H37Rv and acute/chronic MTB HN878 infections. These mice exhibited reduced lung bacterial burdens and alterations in APC responses (107). In addition, during acute MTB HN878 infection, IL-4I1^-/-^ mice exhibited a significant increase in the numbers of “M1-like” interstitial macrophages, as well as higher NO and IFN-γ production when compared to wild-type mice (108). It is possible that the simultaneous deletion of both IL4i1 and IDO1, despite their overlapping expression patterns in myeloid cells, may be necessary to disrupt the three primary biochemical outcomes: tryptophan depletion, AHR activation, and ferroptosis suppression (102, 109).

## Targeting tryptophan catabolism as host-directed therapies of human tuberculous

MTB is naturally prototrophic for tryptophan, meaning it has the ability to synthesize its own tryptophan. Interestingly, auxotrophic mutants of MTB, which are unable to synthesize their own tryptophan and depend on external sources, cannot establish an infection in mice. This emphasizes the essential role of tryptophan biosynthesis for the bacterium’s survival and its ability to cause disease (110). Moreover, studies show increased catabolism of tryptophan (Trp) to kynurenine (Kyn) not only in active TB disease but also during latent TB infection (LTBI) (11). As a result, the Kyn/Trp ratio has been proposed as a potential marker for predicting the prognosis of pulmonary tuberculosis (12). This suggests that monitoring Kyn and Trp levels in individuals could offer valuable insights into the progression and severity of TB. Persons with active TB and LTBI also show an increased expression of IDO1 suggesting that it mediates increased tryptophan catabolism (11, 12). Additionally, systems analysis of the gene and protein expression in human alveolar macrophages from MTB-infected individuals identified a robust network with IL-1β, STAT1 and IDO1 as the hub genes associated with MTB growth and their macrophages shift from an initial M1 to later M2 gene expression with inter-individual variability (13).

It is worth noting that depleted tryptophan resulting from tryptophan catabolism does not significantly affect the growth of MTB since it relies on its own biosynthesis system to produce tryptophan. In this context, elevated levels of IDO1 activity or IDO1 protein have little direct effect on MTB growth and ability to cause disease. Instead, low IDO1 activity has been used as a predictor of death from MTB infection although the mechanisms underlying this inverse relationship are poorly understood. One potential mechanism could be the immunomodulatory effect of kynurenine which is a ligand for AHR. In this scenario, activation of AHR can promote the polarization of macrophage from an M1 to an M2 phenotype where a switch occurs from pro-inflammatory IL-6 and IFN-α to anti-inflammatory cytokines IL-10 and TGFβ. Latter increases the disease severity during pulmonary tuberculosis (111–113). As tryptophan serves as the source of NAD^+^ *de novo* biosynthesis, any excess NAD^+^ generated from the activated tryptophan catabolism seems carefully balanced by multiple regulatory mechanisms. Firstly, NAD^+^ experiences increased hydrolysis and consumption, while simultaneously, NAD^+^ synthesis through the salvage pathway is diminished due to the suppression of NAMPT expression (114, 115). Moreover, the heightened NAD^+^ production exerts limitations on the redox reactions taking place in the mitochondrial electron-respiration chain complex I and II. These reactions generate reactive oxygen species (ROS), which are lethal to MTB. Inhibiting NAMPT activity also results in a reduction in glycolysis, particularly at the glyceraldehyde-3-phosphate dehydrogenase step (116). Meanwhile, tuberculosis necrotizing toxin (TNT) operates by hydrolyze cellular NAD^+^ into NAM (nicotinamide) and ADPR (adenosine diphosphate ribose), thereby activating the necroptosis effectors MLKL and RIPK3 (117). However, an increase of the NAD^+^ hydrolysis products, NAM and ADPR, can competitively impede the TNT-induced respiratory bursts. These bursts often result in an overproduction of ROS, which, in turn, can be detrimental to macrophages. In summary, the complex interplay between NAD^+^ metabolism and tryptophan catabolism seems to play a critical role in macrophage polarization that needs additional research.

The reversal of tryptophan catabolism holds promise as an effective treatment strategy for both active TB disease and LTBI through host-directed therapies. In support of this concept, macaques subjected to treatment with IDO1 inhibitor 1-methyl-DL-tryptophan (1-MT) exhibited reduced mycobacterial burden and lung pathology in comparison with control groups following infection with MTB CDC1551 (107). However, it is worth noting that gene knock-out of IDO1 did not yield consistent results with IDO1 inhibition. Specifically, there was no significant difference in anti-mycobacterial burden observed between IDO1^-/-^ and wild-type mice (118). Conflicting results have also emerged in studies involving AHR, as Ahr^-/-^ mice exhibited an increased mycobacterial burden. The ablation of AHR led to diminished expression of cytokines IL23A and IL12B, which encode subunits of IL-23, a cytokine produced by macrophages that in turn, stimulates the production of IL-22 by innate lymphoid cells (119, 120). Since the impact of tryptophan catabolism on macrophage immunity against MTB infection is intricately linked to various interconnected metabolic processes, a comprehensive approach involving metabolomics, proteomics, and functional studies in the context of MTB infection of macrophages appears warranted.

## Concluding remarks and prospectives

Arginine and tryptophan are two critical amino acids that the immune system utilizes to create both immune-activating and immunosuppressive products. The metabolism of these amino acids plays a crucial role in defending against pathogen invasions and repairing damage caused by infections.

Arginine metabolism is known to have a dichotomous role in immune cells. In IFN-γ stimulated M1 macrophages, arginine is metabolized into citrulline by iNOS, leading to the production of NO and other reactive-nitrogen-species (RNS) that contribute to pathogen killing. In contrast, IL-4-induced M2 macrophages metabolize arginine into ornithine, which serves as a precursor for polyamines to mitigate hyper-inflammation and tissue damage caused by over-activated macrophages.

Tryptophan catabolism, on the other hand, does not exhibit such a clear-cut dichotomy. It has three different metabolic routes leading to the production of kynurenine by IDO1, indole-3-pyruvate by IL4i1, and serotonin, generating downstream metabolites from each pathway. IDO1 is the most extensively studied route due to the immunosuppressive properties of its product, kynurenine, and its role as an AHR agonist. IL4i1 also contributes to tryptophan metabolism, and its metabolic products, such as indole-3-pyruvic acid and its downstream derivatives are AHR agonists with immunosuppressive functions. The expression of both IDO1 and IL4i1 can be induced by IFN-γ. This overlapping expression of IL4i1 and IDO1 in activated macrophages and immune cells highlights the complexity of tryptophan metabolism in immune responses. While both enzymes can lead to the generation of immunosuppressive molecules, their specific roles and regulation may vary in different contexts or after different immune challenges. Contemporary research on tryptophan catabolism in immune responses to MTB infection and the development of anti-TB drugs primarily centers on the IDO1 pathway. However, future investigations should expand to encompass not only the IL4I1 pathway but also the serotonin pathway, in addition to the link of tryptophan catabolism to NAD^+^ metabolism.

**Figure 4:**
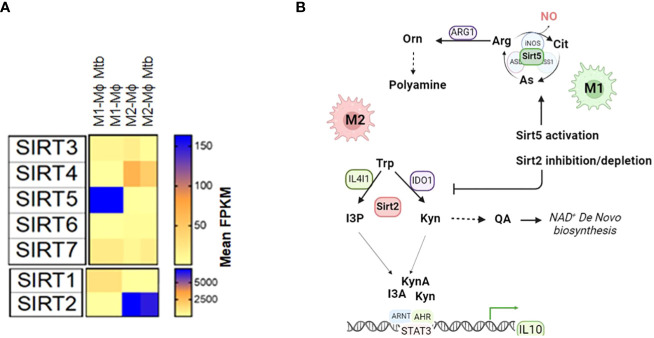
A hypothetic model shows that Sirt2 and Sirt5 regulate the tryptophan metabolism and arginine metabolism to drive macrophage polarization **(A)** Expression of sirtuin proteins (Sirt1-7) in M1-Mϕs and M2-Mϕs infected with and without MTB. **(B)** The hypothetical model of activation of Sirt5 or inhibition/depletion of Sirt2 promotes polarization of macrophages towards M1-Mϕs. Arg, Arginine; Cit, Citrulline; As, Arginosuccinate; NO, Nitric Oxide; Orn, Ornithine; Trp, Tryptophan; Kyn, Kynurenine; QA, Quinolinic Acid; I3P, Indole-3-Pyruvic Acid; I3A, Indole-3-Acetic Acid; KynA, Kynurenic Acid; AHR, Aryl hydrocarbon receptor; ARNT, Aryl Hydrocarbon Receptor Nuclear Translocator; STAT3:Signal Transducer And Activator Of Transcription 3; IL10, Interleukin 10; ETC, electron transport chain; FPKM, Fragments Per Kilobase of transcript per Million.

We propose that Sirtuin proteins may be involved in arginine and tryptophan metabolism. Among the seven sirtuin proteins (Sirt1-7), Sirt2 and Sirt5 are particularly relevant in the context of amino acid metabolism in the immune cells. Sirt2 is upregulated in M2 macrophages compared to M1 macrophages and upregulated in MTB-infected macrophages compared to uninfected ones (1). Sirt2’s role in NAD^+^-biosynthesis from tryptophan-IDO1 metabolism and its connection to the NAD^+^ salvage pathways are areas of interest. Depleting Sirt2 drives macrophage polarization from M2 to M1 (**Figure 4A**). In contrast, Sirt5 is upregulated in M1 macrophages compared to M2 macrophages but downregulated in MTB-infected macrophages compared to uninfected ones. Sirt5 is known to modify proteins related to the extended arginine metabolism cycle that includes glutamine metabolism and glutamate acetylation. Activation of Sirt5 by small molecules like MC3138 may influence the arginine metabolism in favor of iNOS activation and NO production, which is dominant in M1 macrophages (**Figure 4B**).

To gain a comprehensive understanding of how sirtuin proteins shape arginine and tryptophan metabolism, multi-omics approaches, including metabolomics and proteomics, need to be used. These studies, in combination with functional assays, can reveal how sirtuin inhibitors or sirtuin-depleted cell lines impact metabolism (19). Moreover, mechanistic studies are needed to explore how histone lysine acetylation and arginine methylation, influenced by sirtuin and PRMT proteins, regulate gene expression related to autophagy. Overall, understanding the molecular mechanisms of arginine and tryptophan amino acid metabolism and its regulation by NAD^+^- dependent sirtuin proteins can pave the ways for the development of novel host-directed therapy (HDT) drugs for the treatment of tuberculosis, either alone or in the combination with existing anti-TB medications.

## Author contributions

KZ: Conceptualization, Writing – original draft, Writing – review & editing. AM: Writing – review & editing. CJ: Conceptualization, Funding acquisition, Writing – review & editing.
